# From Human Perception of Good Practices to Horse (Equus Caballus) Welfare: Example of Equine-Assisted Activities

**DOI:** 10.3390/ani14172548

**Published:** 2024-09-02

**Authors:** Marine Grandgeorge, Noémie Lerch, Alizée Delarue, Martine Hausberger

**Affiliations:** 1 CNRS, EthoS (Éthologie Animale et Humaine)—UMR 6552, Univ Rennes, Normandie Univ, F-35000 Rennes, France; noemie.lerch@orange.fr (N.L.); alizee.delarue@agrocampus-ouest.fr (A.D.); 2 CNRS, Université de Paris Cité, UMR 8002, Integrative Neuroscience and Cognitive Center, F-75270 Paris, France; martine.hausberger@cnrs.fr; 3 Department Zoology and Entomology, Rhodes University, Makhanda 6139, South Africa

**Keywords:** equid, equid management, equine-assisted interventions, equine working conditions, human–horse relationship, survey, welfare

## Abstract

**Simple Summary:**

Equine-assisted intervention (EAI) studies deal with clients, whereas very few studies focus on the effects on animals. To explore that, this study compared manager’s subjective views of “good” management and selection criteria for a horse in EAI, using an online survey (n = 51) and objective observations in eight additional facilities. Management decisions and welfare consequences were correlated. One major factor influencing welfare and human–equid interactions appeared to be working modalities, with more EAI facilities practicing groundwork and bitless work. Facilities where equids were the most involved in mixed activities (EAI and classic riding school) had the most equids with compromised welfare. Given EAI clients’ particularities, conventional working modalities are less adapted and at risk of increased discomfort for equids. Overall, survey and observational approaches converged but some discrepancies (choice of equid type) appeared.

**Abstract:**

Equine-assisted intervention (EAI) studies deal with clients, whereas very few studies focused on the effects on animals. EAI equids are also submitted to management, which influences their welfare. Management and working conditions depend on human decisions and perception. We gathered information through a survey about facilities managers’ strategies (n = 51) and obtained direct information on management and working practices and their consequences on equids’ welfare through an observational study (n = eight facilities, 174 equids). Differences in managers’ perceptions of good management practices were related to the facility’s involvement in EAI, e.g., increased awareness of equids’ needs (housing and feeding), especially when EAI was the main activity. A detailed observational study on eight additional facilities confirmed that. Facility management profiles were paralleled by equids’ welfare profiles. Clear correlates were found between management decisions and welfare consequences. One major factor influencing welfare and human–equid interactions appeared to be working modalities, with more EAI facilities practicing groundwork and bitless work. Facilities where equids were the most involved in mixed activities had the most equids with compromised welfare. Given EAI clients’ particularities, conventional working modalities are less adapted and at risk of increased discomfort for equids. Overall, survey and observational approaches converged but some discrepancies (choice of equid type) appeared between the reported and observed prevalence.

## 1. Introduction

At a time when animal welfare is an important societal issue, domestic horses constitute a paradox where they are mostly considered as “partners” or companions [1] and, therefore, worth the highest possible welfare conditions. Meanwhile, all welfare studies about horses converge towards a high prevalence of welfare problems [2,3,4,5]. Therefore, assessing managers’ perceptions of good practices and their influence on the actual welfare state of the animals constitutes a very interesting issue.

Animal-assisted interventions (AAIs) are defined by the International Association of Human–Animal Interaction Organisations (IAHAIO) as “goal oriented and structured interventions that intentionally include or incorporate animals in health, education and human services (e.g., social work) for the purpose of therapeutic gains in humans”. Such activities have become increasingly popular, with the species most concerning AAI being first dogs and then equids, e.g., [1]. Over the last decades, the perspectives on AAI animal status has changed from “tools” to individuals with sentience [6]. In a literature review based on 1194 scientific publications on AAI, Michalon and collaborators [1] highlighted that until the 1980s, animals were generally considered as objects (as “detached variables”). In the 1980s, they were given a role as “medicine” on the basis of their therapeutic benefits. It was not until the 1990s that the terms “animal perception” and “animal relationship” emerged, placing the animal in a so-called “partner’s” position. The recent “One health” concept recognizes that human and animal health are interconnected and includes their environment [6]. Today, most AAI associations recommend selecting animals so that they are suitable for AAI and providing them with living and working conditions promoting their welfare [7]. To date, however, there is a large discrepancy in the scientific literature between the abundant literature on the outcomes of AAI for the human clients and the very scarce literature on the welfare of animals involved in AAI [6,8,9]. This is especially crucial in equine-assisted interventions (EAIs) as these activities are most often performed in horse facilities where, beyond the EAI working activity, equids also experience life conditions and other working activities that influence their welfare state, e.g., [2]. In their scoping review on the welfare of EAI equids, Rankins and collaborators [9] found few scientific studies on the topic with more than half of them measuring only the acute reactions of equids to EAI sessions. Only two were devoted to the chronic effects of EAI on the equids’ behaviors outside sessions, both concerning the reactions of EAI equids in a human–horse relationship test. The outcome of both studies was similar: EAI equids were less interactive than riding school or sport equids [10,11]. This suggests that there are indeed chronic effects of the activity that may add to other management factors, but it also shows that there is very little information on the welfare of EAI equids when considering the definition of welfare as “a chronic positive mental and physical state resulting from the satisfaction of the animal’s behavioural and physiological needs and expectations. This state can vary according to the perception of the situation by the animal” [12]. AAI animals must be seen as the least controlling participant in their conditions of life and work [6]. In EAI, the management of facilities is decided by the facility manager, and the working activity either by a horse professional, a therapeutic professional, or both [13]. This leads to very variable situations, with a large variety of activities (e.g., riding, groundwork, harnessing) and a large variety of people (e.g., mental/physical disabilities, social disorders, ageing) but also of equids’ life conditions in terms of housing, feeding, or social situations [14]. Facility managers are in charge of the selection of equids and their overall management; therefore, their choices are decisive for the equids’ welfare. As managers of facilities proposing EAI can first come from two major training lines, conventional riding teaching or therapeutic training, one can expect differences in their perception on how horses should be managed and should work.

Indeed, there is a paradox in the working equids’ welfare status, as most horse owners view them as companions and want the best for them but often do not apply the actual knowledge on horses’ fundamental needs, leading to a generally very poor situation in terms of equid welfare [2,15,16]. In a recent literature review, Hausberger and collaborators [17] identified some major reasons as to why such a discrepancy occurs, amongst which is the “equestrian/barn culture” that exerts a clear social pressure on the way horse owners manage their animals. In Hockenhull and collaborators’ study [18], some horse owners reported having given up on changing their management practices because of social pressures.

More recently, several studies based on surveys, interviews, or workshops have further illustrated how managers of facilities or private horse owners perceive horse welfare and the societal obstacles to achieve the provision of good welfare conditions [9,10,11,12,13,14,15,16,17,18,19,20,21,22]. Of course, the way horse owners conceptualize horse welfare influences the care and training they provide [23], but there have been few studies at the system’s scale (rather than individual factors of influence) [22]. These studies mostly rely upon surveys, e.g., [13,24], which focuses on EAI equids in the USA. Both studies converge in reporting that EAI equids tended to be older (16–20 years, i.e., second or third career as a working equid) than the average working equids, and most had been donated and had fewer health or behavior (stereotypies) problems than the national average apart from back problems. Both mentioned a selection of EAI equids either on temperament (especially behavior towards humans) or sex (geldings perceived as calmer). In Rankins and collaborators’ study [13], only 5% of the respondents reported using their equids only for EAI; all others reported using the equids for different activities.

Whereas surveys prove very useful in order to have a better knowledge of the subjective perception of equid welfare and management by respondents, there is a crucial lack of objective information on the actual welfare status of these equids. Indeed, there are regular discrepancies between reported and observed welfare issues, e.g., [25,26], which is logical given the discrepancy between the cognitive and operational knowledge, the mixing of good horse care and good horse welfare, and the overall lack of attention to the mental health of equids by horse managers reported in a variety of studies [16,22,27,28].

The aim of the present study has been, therefore, (1) to give an overview, through a survey, on how equid facility managers report their management strategies and how this can be influenced according to the proportion of EAI activity in the facility, and (2) to assess, through a detailed observational study at the system level performed on eight additional facilities, how such human decisions influence the equids’ welfare.

**STUDY 1: ONLINE SURVEY**

## 2. Materials and Methods

### 2.1. Ethical Statement

The online survey was conducted in accordance with the Regulation (EU) 2016/679 of the European Parliament and of the Council of 27 April 2016 on the protection of individuals with regard to the processing of personal data and on the free movement of such data and published in the *OJEU* L 119 of 4 May 2016. It complied with the French laws in force relating to information technology, files, and freedoms (Decree No. 2019-536 of 29 May 2019 taken for the application of Law No. 78-17 of 6 January 1978, published in the *OJFR* of 30 May 2019). The survey was registered by the Data Protection Officer of the University of Rennes 1 in the CNIL register (registration certificate 2-19004\UMR6552). Participants in the survey were fully informed of the purpose and context of the study and checked a box to acknowledge having read the general conditions of use. The questionnaire was completely anonymous, and participants could stop the completion of the data at any time.

### 2.2. Survey

#### 2.2.1. Survey Development

Different aspects were considered for the conception of the survey (Table 1 for an extract; see complete survey in Supplementary Table S1):

- Activities: four equestrian activities were considered—“conventional instruction” (riding school lessons) (RS), equine-assisted interventions (EAIs, i.e., activities with people with disabilities such as equine-assisted therapy and riding for the disabled), tourism (i.e., outdoor leisure horse riding, for at least 50 days of riding/year), and boarding (i.e., private horses kept on the facility). Only facilities that proposed at least EAI or RS were kept for this study. A large part of them had both activities (EAI–RS).
- Equids’ management (Table 1): the criteria studied here were based on those that were known to have a particularly important effect on animal welfare, e.g., [2,29]—type of housing (e.g., indoor or outdoor (or both)/singly or in group) and feeding conditions (e.g., quantity of hay/pellets).
- Criteria for equids’ selection for the activity: an open question where managers were asked to describe their selection criteria according to the intended activity of the equids.

The survey was tested and validated by two managers of equine facilities before being put on line to ensure that the questions were understood and that it was easy to fill it out correctly.

Shetland ponies were excluded, however, as the authors’ personal observations were that Shetlands tended to be managed differently from other breeds in most riding schools, with a higher prevalence of group housing and exclusive hay feeding. Therefore, the survey considered only other horse and pony breeds.

#### 2.2.2. Survey Structure

The survey was divided into six parts (see Supplementary Table S1).

The first part was focused on the description of the horse population and of activities: number of horses and ponies (excluding Shetland ponies) and activities proposed (i.e., riding school lessons, EAI, tourism, and boarding). Depending on their answers, the participants had access to parts 2 to 5. Part 2 was about the equids involved in riding school lessons, part 3 on equids involved in tourism, part 4 on EAI, and part 5 on boarded, private equines. They started with an open question about their selection criteria for the equids involved in the activity, which was followed by questions on work routines (i.e., the number of sessions per week and the duration of sessions), and finally by questions on living conditions (see Table 1 for details). In order to limit biases, we followed the recommendations proposed in a recent review on horse management questionnaires [30]. Thus, we chose to ask closed questions with a neutral choice of answers, describing simply the different existing management practices.

Finally, part 6 concerned informative data about the facility and respondent such as the year of birth, gender, and role of the respondent in the facility as well as region/location of the facility.

### 2.3. Respondents

The survey was kept online between 1 March 2021 and 30 June 2021 on the Limesurvey platform. It was spread via mailing lists and social networks (Fondation Adrienne et Pierre Sommer, IFCE—Institut Français du Cheval et de l'Équitation, FNC—Fédération Nationale du Cheval, as well as the list of former professional students who participated to Rennes University Degree «Ethologie du Cheval»). It was also sent to managers of equestrian facilities throughout France. No other inclusion criteria were required.

A total of 216 persons responded to the survey. Among them, 130 respondents had to be excluded because their responses were incomplete. Out of the remaining 86 responses, 35 were from equestrian boarding or tourism facilities that did not offer any of the activities requested for the present study (RS or EAI) and were not analyzed here. Thus, a total of 51 responses were kept for the present study. The respondents were 46 women, 6 men, and 5 persons who did not provide this information, with an average age of 46.9 ± 2.1 years (15 NA) at the time of the study. The facilities they managed were located in 36 different French counties (named French départements) in mainland France. The activities proposed in each facility were recorded (Table 2).

### 2.4. Data Analyses

Responses from different facilities proposing the same activities were grouped in order to calculate the percentages of answers to the different choices for each question. Furthermore, responses for riding school (RS)-lesson equids were compared according to whether the facilities proposed additional EAI or not. Similarly, responses for EAI equids were compared according to whether the facilities also proposed RS or not.

As the questions concerning the selection criteria of equids were open questions, we classified them into different categories: intrinsic (i.e., genetic) individual characteristics (subcategories: age, sex, and physical characteristics), health (mental or physical), background (past living conditions and past working conditions), the level of schooling, personality (emotionality, activity level, horse–human relationship, other personality traits [31]), and no criteria.

Finally, since these were open-ended questions, the answers were coded by qualitative thematic analysis based on either post-hoc thematic choice.

### 2.5. Statistical Analyses

All statistics were performed using R software (version 4.0.2; R Core Team, 2018). Fisher exact tests were performed to assess if there were differences in management according to the activities of each facility, both for EAI equids (with or with no RS) and for RS (with or with no EAI). Finally, the proportion of facilities within each group of activities that mentioned the given criteria in each category was compared according to that of other groups of facilities (see above).

## 3. Results

### 3.1. Responses about Equids’ Management

#### 3.1.1. Management of Riding-School-Lesson Equids (Table 3)

More than half of the respondents from facilities proposing riding school lessons indicated that their equids were kept permanently in pasture or paddock; around a quarter reported that most of their equids were in alternance between paddock and stall, and 18.2% reported that they kept them in a permanent stall housing. Half reported that their equids were always kept in a group (collective stall, paddock, or pasture), a quarter of them that their equids spent more than 3 h/day in a group and the rest of the time singly, and 20.5% that their equids were maintained in permanent single housing (individual stall or individual paddock). More than half also reported giving more than 9 kg of hay per day (i.e., when grass was not available or as an addition) per equid, 36.4% 3 kg to 9 kg, and fewer, 0 to 3 kg hay per day. Most respondents reported giving pellets or cereals, amongst whom a third reported giving 2 meals per day; for a quarter, 3 or more meals per day; for 18.2%, 1 meal per day. Thus, 20.5% reported giving neither pellets nor cereals.

However, differences appeared according to whether the facilities did or did not propose EAI in addition to RS: more respondents from facilities without EAI reported giving between 0 and 3 kg per day (Fisher exact test: *p* = 0.029). No difference appeared for the other parameters (housing: *p* = 0.138; social contact: *p* = 0.090; number of pellet meals per day: *p* = 0.598; number of meals of hay per day: *p* = 0.365).

#### 3.1.2. Responses on the Management of Equine-Assisted Intervention Equids (Table 3)

Most managers of facilities including EAI reported keeping their equids outdoors (pasture or paddock), 18.8% reported keeping them indoors (stall), and 12.5% reported keeping them in mixed housing with at least 3 h outdoors per day. Most reported keeping them always in a group, while 28.1% reported keeping them permanently in single housing (single stall or paddock) and 12.5% in a group for more than 3 h a day. More than half reported giving permanent access to roughage, and only 2 reported no permanent access. Almost half of the respondents reported giving between 3 kg and 9 kg, 12.5% reported giving less than 3 kg per day, and 40.6% reported more than 9 kg per horse or pony when grass was not available or as an addition.

Forty percent reported giving no pellet, 21.9% one pellet meal, 12.5% two pellet meals, and a quarter at least three pellet meals per day.

**Figure 1:**
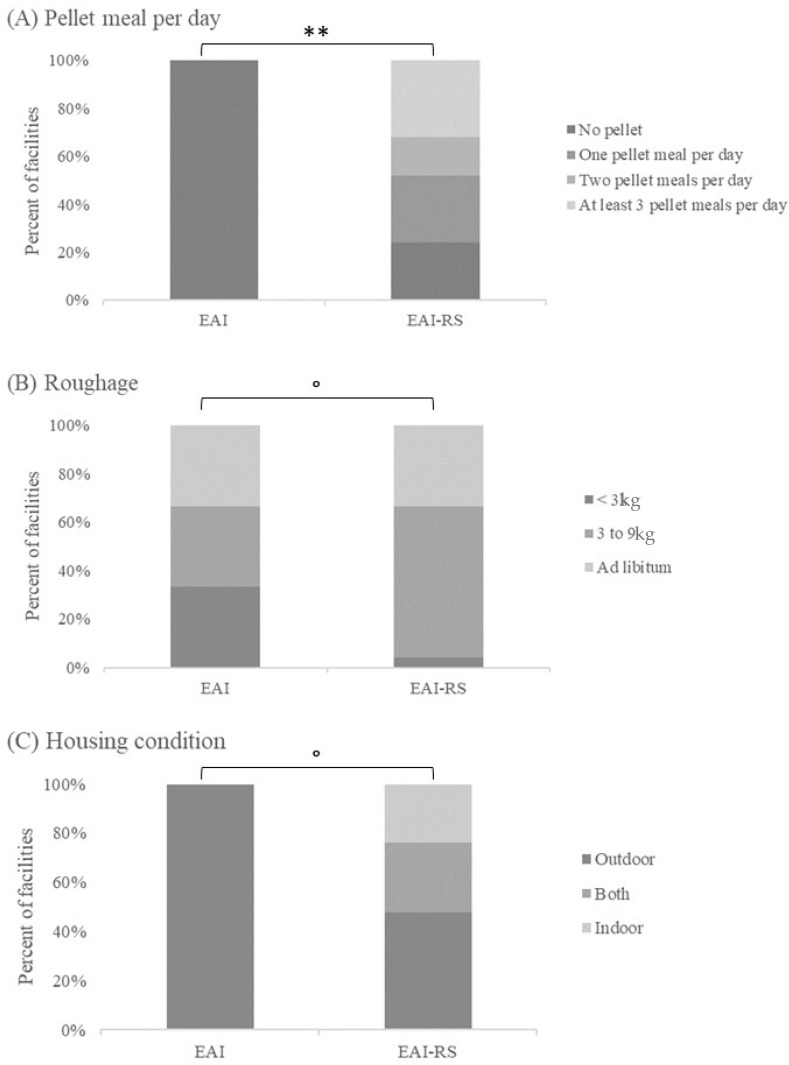
Comparison of reports on management conditions according to whether the respondents managed facilities with EAI activities only or EAI and RS activities: (**A**) pellet meal per day, (**B**) roughage availability, and (**C**) housing condition. Fisher exact test, ** *p* < 0.01, ° tendency: *p* < 0.06. EAI: equine-assisted intervention; RS: riding school.

Differences appeared in the responses according to whether the facilities proposed riding school lessons in addition to EAI or not, regardless of the other activities. Managers of EAI facilities that did not offer riding school lessons reported feeding their equids with less pellets (*p* = 0.005; Figure 1A) and more roughage (*p* = 0.057; Figure 1B), and they tended to house them more in the outdoors (*p* = 0.057; Figure 1C) than managers of EAI facilities that also proposed riding school lessons.

#### 3.1.3. Management in EAI–RS Facilities

The responses of managers of facilities proposing both EAI and RS reported a similar management for EAI and RS equids (Fisher exact test: housing condition: *p* = 1; social contact: *p* = 0.817; number of pellet meals per day: *p* = 0.583; number of meals of roughage per day: *p* = 0.956; quantity of roughage per day: *p* = 0.660).

### 3.2. Criteria for the Choice of Animals (Figure 2)

#### 3.2.1. Respondents from Facilities Proposing Riding School Lessons

Most managers (68.2%) (n = 30) reported horse personality as an important criterion (36.4% emotionality (n = 16), 29.5% (n = 13) relationship to humans, 6.8% (n = 3) activity level, and 20.5% (n = 9) other traits). The second aspect (61.5% of the respondents) (n = 27) concerned other intrinsic characteristics (56.8% morphological characteristics (n = 25), 9.1% age (n = 4), and 2.3% sex (n = 1)). Half of the respondents (50.0%) (n = 22) mentioned the horse or pony’s level of schooling. Twenty-five percent of the respondents (n = 11) reported considering the equid’s past experiences (22.7% past living conditions (n = 10) and 2.3% past working conditions (n = 1)). Sixteen percent (n = 7) mentioned health (9.1% physical health (n = 4) and 6.8% mental health (n = 3)), and finally 13.6% of the respondents (n = 6) said that there was no need for selection criteria.

**Figure 2:**
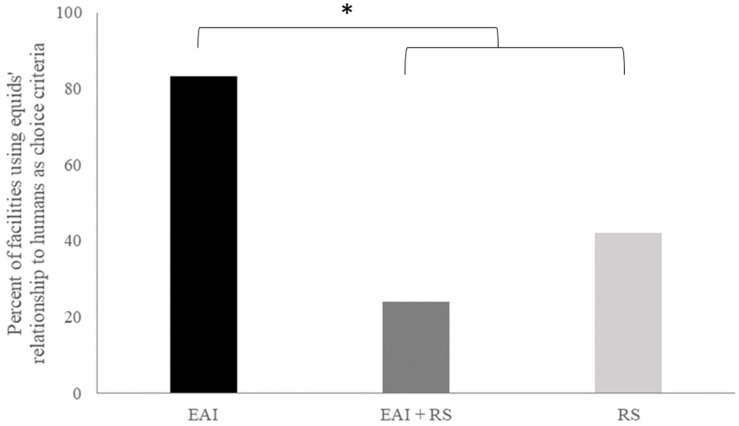
Percent of facilities using equids’ relationship to humans as criteria for choosing them, according to their activities (EAI only, EAI–RS, or RS only). Fisher exact test: * *p* < 0.05. EAI: equine-assisted intervention; RS: riding school.

Differences appeared between responses of managers according to whether the facilities did or did not propose EAI in addition to RS. Only in the latter case, RS managers reported that they had no selection criteria (Fisher exact test: *p* = 0.004). No difference was found for the other criteria (personality: *p* = 0.327 [relationship to humans: *p* = 0.182; emotionality: *p* = 0.113; activity level: *p* = 0.247; other: *p* = 0.26]). No difference was observed either for other intrinsic characteristics (all characteristics grouped: *p* = 0.76 [sex: *p* = 1; age: *p* = 0.744; morphology: *p* = 0.361]; health: *p* = 0.680 [physical: *p* = 1; mental: *p* = 0.247]; past: *p* = 1 [living conditions: *p* = 0.723; working conditions: *p* = 1]; level of schooling: *p* = 0.22).

#### 3.2.2. Facilities Devoted to Equine-Assisted Interventions

Most (83.9%) (n = 27) respondents reported choosing according to personality (51.6% emotionality (n = 14), 37.0% relationship to humans (n = 10), 6.5% activity level (n = 2), and 22.6% other character traits (n = 6)) and 43.7% (n = 14) mentioned other individual characteristics (41.9% physical characteristics (n = 6), 7.1% sex (n = 1), 7.1% age (n = 1)). One third (32.3%) (n = 10) mentioned the level of level of schooling and a quarter (25.8%) (n = 8) the health state (6.5% physical health and 19.4% mental health). Some (16.1%) (n = 5) mentioned the past life of the animal (16.1% past living conditions and 3.2% past working conditions), and, finally, 3.2% (n = 1) said they had no selection criterion.

Differences in the responses appeared according to whether the EAI facilities proposed or not riding school lessons as well. Respondents from EAI facilities that did not propose riding school lessons reported more often the equids’ relationship with humans as a selection criterion (*p* = 0.013) (Figure 2). No difference was found according to the type of activity proposed for the other criteria (other individual characteristics: *p* = 0.664; [sex: *p* = 1; age: *p* = 1; morphological: *p* = 0.1]; health: *p* = 0.161 [physical: *p* = 1; mental: *p* = 0.069]; past living or working conditions: *p* = 0.241; level of schooling: *p* = 0.634; character: *p* = 1 [emotionality: *p* = 0.083; activity level: *p* = 0.355; other personality traits: *p* = 0.11] and no criterion: *p* = 1).

#### 3.2.3. Comparison of Responses from Managers of Mixed Facilities According to Equids’ Activities

Managers reported no difference in terms of equids’ choice criteria according to their intended activity (Fisher exact test, EAI versus RS equids: personality *p* = 0.725 [relationship to humans: *p* = 1; emotionality: *p* = 0.571; activity level: *p* = 0.609; other: *p* = 0.496), other individual characteristics: *p* = 0.393 [sex: *p* = 1; age: *p* = 1; physical: *p* = 0.256]; health: *p* = 1 [physical: *p* = 1; mental: *p* = 1]; past: *p* = 0.464 [living conditions: *p* = 0.702; working conditions: *p* = 1]; level of schooling: *p* = 1; no criteria: *p* = 1).

## 4. Discussion

The activity proposed by the facilities seemed to have a great influence on how the respondents reported managing their equines. Thus, there was more stall/mixed housing, more facilities giving less than 3 kg hay per day (the only roughage available when housed in stall or stall/paddock housing), and more feeding of pellets in the facilities proposing riding school lessons, especially when they proposed only riding lessons and not EAI as well (see Figure 1). Managers of facilities proposing only EAI reported more outdoor group housing and less pellet feeding. Although it could be argued that the higher use of pellets for feeding the equids in RS facilities may be due to their (perceived?) increased need of calories because of a harder workload, there is no evidence that their work is indeed harder as the emotional/physical constraints of EAI may be high [8] nor evidence that there was a need for the three or more meals per day as declared for 25% of the managers. Excess energy intake is common in working equids of developed countries leading to digestive and other chronic health problems, e.g., [2]. In mixed facilities, managers reported managing EAI and RS equids the same way. Most respondents reported choosing equids on the basis of personality, while physical characteristics and education level came next. However, here again, differences appeared depending on the activities proposed by the facilities: managers of RS facilities that did not offer EAI were more likely to report having no selection criteria, whereas those of EAI facilities that did not offer an RS reported more choosing equids on the basis of their relationship with humans. This first study clearly showed that the perception of equine management by stakeholders was particularly influenced by the “equestrian culture”, which resulted in a divergence in the management of EAI equids according to the managers’ culture. Indeed, managers of “conventional” riding school facilities did not seem to change their equids’ management when they were also involved in EAI activities.

Overall, the reports on this survey suggested a more accurate awareness of managers of EAI facilities of horses’ needs in terms of housing, social, and feeding conditions, e.g., [29], than earlier observational and survey studies found in other European riding school facilities where single-stall housing and restricted hay provisioning predominate, e.g., [2,3,32]. The selection of horses on the basis of their relationship to humans has been reported repeatedly in earlier studies too by EAI practitioners, although this was not clearly validated by experimental tests or observations, e.g., [33].

It has to be noted that this study has some limitations. First of all, even if our sample size is quite important, one could argue that it may be not fully representative of the whole equine practice and that it can be biased by the fact that professionals who are more aware of the fundamental needs of equines were more likely to respond to such a survey. Moreover, discrepancies between reports in surveys or direct questionnaires and practices are common either because of expectations on the social acceptability of responses, discrepancies between public and personal claims, or conflict between cognitive and practical knowledge or simply the lack of knowledge [16,17,25]. Therefore, direct observational studies are absolutely necessary to better understand how facilities are really managed and what consequences the managing decisions have on horse welfare.

**STUDY 2: On-farm observational study**

Study 2 was devoted to direct observations of management practices in eight riding schools (not included in the survey) proposing EAI as a main or secondary activity and to their consequences in terms of equine welfare.

## 5. Materials and Methods

### 5.1. Ethical Statement

The experiments were carried out between February 2019 and December 2020 in accordance with the Directive 2010/63/UE of the European Parliament and the Council on the protection of animals used for scientific purposes. They complied with the current French laws related to animal experimentation (decree n° 2013 ± 118 of 1 February 2013) and its five implementation orders, JO 7 February 2013, integrated into the Rural Code and the Code of maritime fishing (under n° R.214 ± 87 to n° R.214-137). The experiments performed in this study were not within the scope of application of the European directive, thus, in accordance with this directive and the current French and Irish laws, the following experiments did not require us to request authorization. These experiments involved only behavioral observations and non-invasive interactions with the horses. The horses used in this research were not research animals. Animal husbandry and care were under the management of the riding school staff. The riding school managers gave the authors their informed consent for this study.

### 5.2. Subjects, Facilities, and Management Practices

#### 5.2.1. Study Sites

Seven French (and one Irish) equestrian facilities, located in a variety of regions, were included in this study (Table 4). Observations were made between February and May 2019 in facilities 7 and 8 and between September and December 2020 in facilities 1 to 6. Facilities 1 and 2 were located in Auvergne-Rhône-Alpes, facilities 3, 5, and 6 in Nouvelle-Aquitaine, facility 4 in Brittany, facility 7 in Grand Est, and facility 8 in Ireland. All these facilities proposed EAI programs for a large variety of clients (i.e., mental and physical disabilities and/or social difficulties). Facilities 4, 5, 6, 7, and 8 proposed also conventional riding school (RS) lessons, and facilities 1, 2, 3, 5, and 6 had private horses in boarding. In each facility, equines involved in EAI, as well as equines not working in EAI, were studied.

#### 5.2.2. Equids’ Characteristics

A total of 174 equids were included in the study. For each equid, the sex, age, and breed were recorded. Shetland ponies were excluded for the same reason as in Study 1. Seventy-seven mares and 97 geldings, aged 4 to 27 years (mean ± SE = 14.1 ± 0.4 yo) and from various breeds but mostly unregistered, were studied. Ponies and horses were distinguished according to the FEI categorization (i.e., pony ≤ 1.48 m at withers or horse > 1.48 m). The morphological category was also recorded based on the proportion between length (from the point of shoulder to the point of buttock) and the height at the withers: dolichomorphic (length > height), mesomorphic (length = height), and brachymorphic (length < height). Indeed, previous studies have shown that horses with longer backs are more likely to suffer back problems [2,34,35]. Finally, in order to test for possible age differences, for data analysis, equids were divided into one of two age classes classically used in horses: ≤15 years and >15 years [2,10,36].

#### 5.2.3. Housing and Feeding Conditions

None of the equids was kept in permanent single stall housing. However, the duration of daily time turnout varied largely between sites, from 30 min to 21 h per day (mean ± SE = 7.2 ± 0.7 h). When outdoors, all equids were in stable groups. The housing practices were generally common to all equids within a facility.

In total, 74 equids were kept permanently or almost permanently (more than 20 h/day) outdoors in collective pastures (i.e., grass present, 57 equids, group size between 2 and 34 equids) or paddocks (i.e., bare ground, 17 equids, one group of 7 and one group of 10 equids); 47 were housed indoors in collective stalls with daily time (>3 h/day), in the pasture (29 equids, groups between 4 and 34 equids), or in the paddock (18 equids, groups between 2 and 4 equids); and 53 equids were housed indoors in individual stalls with daily (or weekly) time in the pasture (32 equids) or in the paddock (21 equids).

All equids had access to water ad libitum and had hay, but not all horses received pellet or cereal meals in addition. Those kept in pastures had access to grass. For data analyses, two categories were considered for hay: ad libitum access (130 equids) or restricted access (2 kg to 6 kg per day, 44 equids). The number of meals of commercial pellets per day was also noted (no meal: 94 equids; 1 meal per day: 8 equids; 2 meals per day: 48 equids; or 3 meals per day: 24 equids).

#### 5.2.4. Working Conditions

A total of 25 equids worked only in equine-assisted interventions (EAIs), 40 in conventional riding school (RS) lessons, 20 were private horses boarded in the facility, and 89 were involved in two activities (EAI–RS). The time spent working per week was recorded for each equid, and two categories were made according to the median number of hours worked per week ([0–5.5 h] and [5.5–14 h] per week). Concerning the working modalities, 19 equids had never been ridden, and 34 never did groundwork. One hundred and thirteen equids worked with a bit, and 51 never wore a bit.

### 5.3. Welfare Assessment

Welfare assessment was made from animal-based measures, i.e., welfare indicators [37]. Since most welfare assessment protocols were defined for animals housed indoors [2,4,38], we chose to use or adapt indicators that could be used for equids both kept in stalls and outdoors.

Moreover, some indicators of compromised welfare such as “depressed” states [36,39] or facing a wall [40], even for those housed in stall, were not observed in the study population.

All observations were performed by one single experimenter (N.L.) who was trained until the total agreement of a senior experimenter (M.H.).

#### 5.3.1. Health-Related Indicators

- Body lesions

All equids were examined and all hairless patches, scabs, skin lesions, wounds, and marks of former wounds (white hair areas) related to the equipment (at equipment contact points: location of the saddle, girth, bit/bridle, spurs, harness) were noted. Subjects were then binary classified: no equipment injury/presence or at least one equipment injury.

- Body assessment

Various studies have shown a high prevalence of overweight or even obesity in riding school horses, which is often under evaluated by managers for review, see [17]. Horse obesity has been correlated with hyperinsulinemia and insulin resistance [41,42]. Here, a Body Condition Score (BCS) was evaluated according to Arnaud and collaborators [43]. Thus, a score out of 5 was obtained for each equid (from 0 = emaciated to 5 = obese). As all equids here were optimal (BCS = 3), fat (BCS = 4), or obese (BCS = 5), subjects were divided into two categories: “optimal” (3–3.5) or “overweight” (4–5).

- Neck shape

Neck shape has proved a useful indicator of spine state in earlier studies, with flat or hollow necklines being associated with more tension along the spine, hence back problems [34,44] and more welfare problems overall [45,46,47]. We followed Lesimple and collaborators’ protocol [44] to characterize neck shape.

#### 5.3.2. Postural Indicators: Ears’ Positions

The time spent with ears backwards while feeding on roughage or grass has been correlated with chronic welfare impairment, such as vertebral disorders or stereotypic behaviors [48,49]. For each subject, 15 ear positions were recorded during different scan-sampling sessions of 30 min (a scan every 5 min), when the equid was feeding on roughage (hay, straw, or grass), following the method previously developed [2]. The scan-sampling session stopped before the 30 min if the animal stopped feeding. During the observation, the experimenter walked slowly and regularly (1 step/second), 2 m away from the stalls in stables or from the edge of the field. Only if the equid kept feeding and paid no attention to the experimenter, the instantaneous ear position of the feeding equid was silently noted. The observer then resumed her walk up to the next subject. Four positions were recorded [2,50]: forward (pinnae only visible from front), backward (pinnae only visible from behind,), sideward (pinnae visible from the side, between backward and forward ears) or asymmetrical (both ears in different positions).

#### 5.3.3. Behavioral Measures

- Stereotypic/abnormal repetitive behaviors (SB/ARB, pooled as “SB”)

SB and ARB are repetitive behavioral sequences, performed with no obvious goal or function and which are considered to reflect chronic stress [51]. They appear in particular when equids are in suboptimal housing [52,53,54] or feeding [54,55] or working conditions [56,57]. The number of SB and ARB was recorded for each equid during three sessions of 10 min following an “all occurrences” sampling [58]. A behavior was considered an abnormal repetitive behavior when it was repeated at least three times in succession and had been observed at least five times regardless of the observation period [25]. The same SB and ARB as in Lesimple and collaborators [2] were recorded, see also [59]:

Weaving: obvious lateral movement of head, neck, forequarters, and sometimes hindquarters;

Cribbing/windsucking: the horse grasps a fixed object with its incisors, pulls backwards, and draws air into its esophagus;

Headshaking: large vertical movements of head and neck;

Head tossing/nodding: vertical movements of head;

Striking with forelimb: the horse hits the door or wall with one of its forelegs;

Stall walking: repetitive tracing of a route within the stable;

Compulsive licking: licking of the same object in its environment (except the trough);

Compulsive biting: biting of the same object in its environment (except the trough);

Head movements (other than head shaking/tossing/nodding): movement of the head;

Vacuum threats: the horse expresses threat sequences (kicking or biting) alone in its stall;

Mouth open: the horse keeps its mouth open with a lateral movement of its neck;

Teeth rubbing: rubbing teeth on the upper part of the door;

Teeth chattering: mouth movement with teeth chattering;

Lips movements: clapping of lips;

Tongue movements: movements of tongue, inside or outside the mouth.

- Reactions to the experimenter in horse–human relationship tests

Equids’ reactions during human–horse interaction tests can be influenced by their welfare state. For example, it has been shown that horses with back problems are more aggressive towards humans [48] or that apathetic horses are less responsive to the approach of an unfamiliar human [36,39]. In this study, three tests classically used to assess the human–horse relationship were performed [60,61]. For these tests, the animals were isolated (but in view of their group members or neighbors) and tested in a familiar environment (stall or part of a pasture).

Motionless person test: while the animal was foraging on the ground (roughage/grass), the experimenter entered and stood facing the animal with her/his back against the door/entrance for 5 min, motionless, with her/his arms at her/his side, without trying to interact with the animal. All the animals’ behaviors towards the experimenter (according to Lerch and collaborators’ behavioral repertoire [10]) were noted continuously.

Approach–contact test: The experimenter stood 1.5 m away from the horse, perpendicular to its shoulder. When the horse started feeding again, the experimenter slowly moved towards it (one step per second) with her/his arms at her/his side until she/he reached the animal’s shoulder, which she/he then tried to touch with her/his hand. The test was performed on one body side, then on the other, in a random fashion. All behaviors of the horse during the approach and at the time of contact were noted. If the animal moved away or threatened the experimenter, the test was considered a failure and was stopped, and a new attempt was made as soon as the animal had started feeding again. After three attempts, the test was stopped on that side.

Saddle test: The experimenter suddenly appeared at the door/entrance while the animal was feeding on the ground and opened it so that the equid could see the saddle. A reaction score was assigned, based on Hausberger and Muller [62]—the reaction received a score of A if the equid approached looking at the human with ears forward, B if the equid looked with ears forward but remained at the same place, C if the equid did not react (no change in activity), D if the equid looked at the human with ears back without moving, and E if the equid approached the experimenter with ears back, threatening to bite or kick.

### 5.4. Data Analysis

Since our research aimed at considering data at the facility level, we calculated for each facility the proportions of equids involved for each measure and compared these proportions between facilities.

All welfare indicators were transformed into categorical variables: the absence or presence of at least one injury or the trace of a former work-related injury; normal BCS or overweight BCS; more than 50% of feeding time with ears forward or less than 50%; more than 50% of feeding time with ears backward or less than 50%; round neck or flat/hollow neck; no SB/ARB or at least one SB/ARB; no human-oriented behavior in the motionless test or at least one human-oriented behavior (whatever the valence, see [10]; negative, positive, or no reaction in the contact-approach test; negative (D or E), positive (A or B), or no reaction (C) in the saddle approach test.

Similarly, for the management parameters, categories were established. When the parameters were continuous, the median was calculated (e.g., number of working hours per week and number of hours outdoors) to obtain the most balanced categories. Thus, the categories studied were: housed 24 h outdoors or less than 24 h outdoors; foraging possibilities (roughage: grass or hay) ad libitum or restricted; at least one meal of pellets or no meal of pellets; more than 5.5 h work or less than 5.5 h work per week; ridden work or no ridden work; ground work or no ground work; work without bit or with bit. The same was carried out for the choice of equids: brachymorph, mesomorph, or dolichomorph; horse or pony; mare or gelding; >15 years or ≤15 years.

Finally, the equids were classified into three categories of activities: EAI (100% EAI); RS (0% EAI); EAI–RS (mixed).

### 5.5. Statistical Analyses

Firstly, in order to highlight profiles, a visual analysis was carried out by calculating radars for each facility. Thus, a first radar was made with the proportion of equids for each management parameter, and a second was made with the proportion of equids for each individual characteristic, thus representing the parameters of the choice of animals by the facilities managers. Lastly, a third radar was made with the proportion, per facility, of equids for each welfare indicator.

All statistical analyses were performed using R software (version 4.0.2) (R Core Team, 2018). Using the catdes function (FactoMineR package) [63] from the data for each individual, each equestrian facility was characterized according to the significantly more represented factors by calculating a test value [64]. The analysis was performed separately for the welfare indicators, management parameters, EAI, and choice of animals. The following analyses were all performed at the facility level, using the proportions of individuals in each category. An exception was made for the time spent outdoors because, as the horses in the category “less than 24 h outdoors” included horses that spent 21 h outdoors and others that spent 30 min outdoors, it was not the proportion of horses that was used but the median time outdoors per facility.

The possible correlates between management practices (as well as the choice of animals) and the welfare state of equids were tested using Spearman correlation tests (as data were continuous).

## 6. Results

### 6.1. Facility Profiles (Figure 3 and Figure 4)

The analysis of the data confirmed the existence of facility profiles in terms of management, the selection of equids, and equid welfare.

#### 6.1.1. Management Profiles

**Figure 3:**
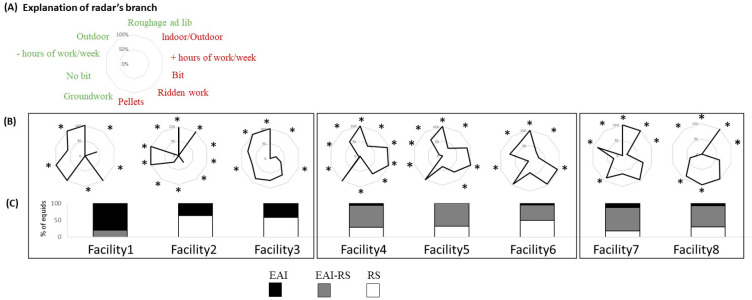
Management profiles of the different facilities (in % of equids per measure, e.g., working with a bit or no bit, % of equids having access to roughage ad lib. or median value, e.g., over or under the median value of 5.5 h per week, see material and methods), with (**A**) categories considered on the management profiles, on the left, parameters considered as “positive” (green), on the right, parameters considered as “negative” (red), both in relation to the existing scientific literature (e.g., [2,29]; (**B**) radars of management per facility, grouped according to the similarity/proportion of “positive” vs “negative” indicators; and (**C**) the proportion of equids involved in EAI activity, RS activity, or EAI–RS activity. * *p* < 0.05 for data statistically different from the average. EAI: equine-assisted intervention; RS: riding school.

There were clear differences between facilities in their management profiles, with three types coming out (Figure 3A,B): (1) facilities 1 to 3 were characterized by a majority of “positive” life and/or working conditions, with more naturalistic life conditions (more outdoors, little or no pellet feeding) and/or lighter work (more groundwork, more bitless, less hours/week); (2) facilities 4 to 6 presented a mix of rather favorable conditions (more hay, outdoor housing, and groundwork) but “heavier” working conditions (more hours/week, more use of bit), (3) facilities 7 and 8 presented the most unfavorable conditions (more indoors housing, more ridden work, more use of bits, more pellets). It is interesting to note that the most appropriate management was observed in the facilities where more horses were involved in EAI (only) activities (compared to mixed or RS activities) (Figure 3C).

Statistical characteristics of the facilities are given in Supplementary Table S2: overall, five facilities (1, 3, 4, 5, and 6) were characterized by a higher proportion of equids living outdoors and having permanent foraging opportunities (grass and/or hay) and three (2, 7, and 8) by most equids living indoors with daily turnout. Three facilities (1, 2, and 4) did not feed on any cereals or pellets. Finally, three facilities (4, 5, and 6) had most equids working with a bit and, in facilities 4 and 5, for more than 5.5 h per week (4 and 5).

#### 6.1.2. Equid Characteristics

In terms of animal population, facilities 5, 6, and 8 showed a large variety of sex, age, and equid types, suggesting that the manager did not have any particular selection criterion (all *p* < 0.05). Facilities 1 and 7 were characterized by a higher percentage of mares than geldings (both *p* < 0.05), facilities 2, 6, and 7 by a higher percentage of horses than ponies (*p* < 0.05), and facility 4 by more ponies than horses (*p* > 0.05). There was no preference in any of the facilities for any age class. Therefore, there was no relationship between the main activity in the facility and any selection criteria (all *p*>0.05), only some individual preferences for one sex over the other or equid type. All facilities had a majority of dolichomorphic equids (all *p* < 0.05) (Supplementary Table S2).

**Figure 4:**
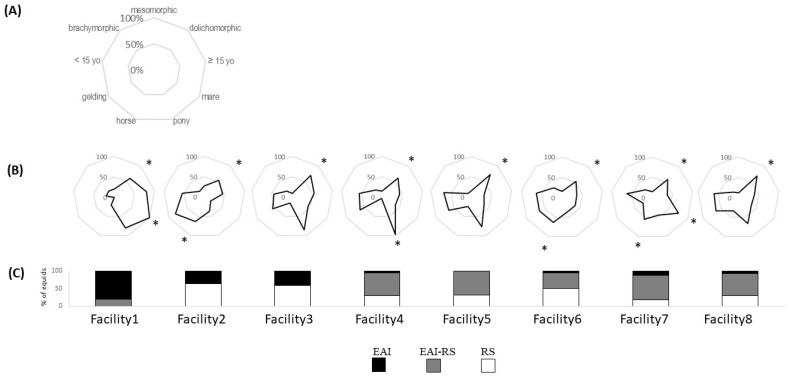
Equids’ characteristics profiles in the different facilities (in % of equids per measure, e.g., % of ponies), with (**A**) equids’ characteristics measured, (**B**) radars of equids’ characteristics per facility; and (**C**) proportion of equids involved in EAI activity, RS activity, or EAI–RS activity in each facility. * *p* < 0.05 for data statistically different from the average. EAI: equine-assisted intervention; RS: riding school.

#### 6.1.3. Equids’ Welfare Profiles

**Figure 5:**
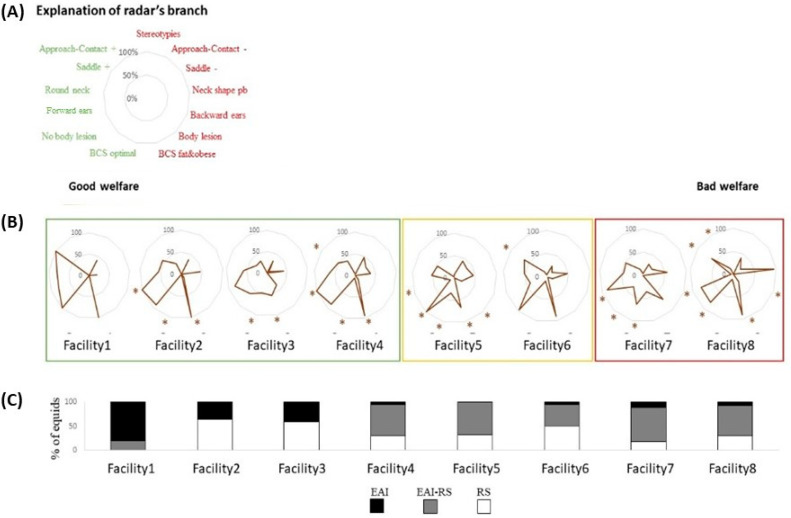
Equids’ welfare profiles in the different facilities (in % of equids per measure, e.g., % of horses with round neck), with (**A**) welfare indicators, on the left, parameters considered as “positive” (green) in relation to the existing scientific literature, on the right, parameters considered as “negative” (red); (**B**) radars of welfare per facility, grouped according to similarity/proportion of “positive” vs “negative” indicators: facilities 1 to 4 showed more general positive welfare states (three positive indicators and only one negative indicator represented by more than 50% of the equids), facilities 5 and 6 had two positive indicators and only one for the negative indicators represented by more than 50% of their equids, facility 7 had two negative indicators and two positive indicators represented by more than 50% of its equids, and facility 8 had two negative indicators represented by 100% of its equids while having three positive indicators represented by around 50% of its equids. For this last category, the proportion + versus − in terms of number and proportion of equids led us to consider here that they corresponded to the “less good welfare” category; and (**C**) proportion of equids involved in EAI activity, RS activity, or EAI–RS activity in each facility. * *p* < 0.05 for data statistically different from the average. EAI: equine-assisted intervention; RS: riding school.

Three main profiles appeared also on the animal-based welfare indicators (Figure 5): (1) facilities 1 to 4 were characterized by more positive indicators but for body score, which tended to be too high for three of them, (2) facilities 5 and 6 presented a “mixed” profile with some positive elements (no equipment-related injuries or positive reactions to the saddle test) but also some negative aspects, (3) facilities 7 and 8 presented more indicators of compromised welfare (e.g., hollow necks in facility 8). Facility 1’s data could not be statistically tested given the low sample, but it is worth noting that equids in this facility were characterized by almost only positive indicators.

Information on facilities and statistical results are in Supplementary Table S2. Facilities 1, 2, and 4 had a large proportion of overweight animals, and facility 5 had a high proportion of animals with at least one work-related skin lesion or the trace of an old lesion. Facilities 7 and 8 appeared to differ from all other facilities with most horses presenting some indicators of compromised welfare with less forward ears when feeding and, in the case of facility 8, with also more negative reactions to humans.

#### 6.1.4. Involvement in EAI and Welfare

There were clear variations in the equids’ welfare measures according to the proportion of horses dedicated to EAI in the facility. Thus, the higher the proportion of EAI equids (i.e., EAI as sole activity), the lower the proportion of equids with flat/hollow necks, hence potential back problems (N = 8, Spearman test, rho = −0.73; *p* = 0.040). In contrast, the higher the proportion of RS equids (i.e., RS as sole activity), the more equids showed no interactive behavior in the saddle test (rho = 0.88; *p* = 0.007).

Finally, the higher the proportion of equids with mixed activities (EAI–RS equids), the higher the proportion of equids presenting at least one type of stereotypy (rho = 0.92; *p* = 0.001) and of equids reacting negatively to the saddle approach test (scores D+E, rho = 0.90; *p* = 0.002).

#### 6.1.5. Management and Welfare

There were clear relationships between management factors and the equids’ overall welfare state in the facility. Management profiles and equid welfare profiles tended to correspond to the same groups of facilities and to follow the same gradient (Figure 3 and Figure 4).

This can be explained by clear correlations between individual management factors and the proportion of horses exhibiting positive or negative welfare indicators within the facilities.

Thus, correlations were found for the following:

- Feeding conditions: The higher the proportion of equids with access to ad libitum foraging, the higher the proportion of non-interactive (no reaction) equids during the motionless person test (rho = −0.80; *p* = 0.032), i.e., the higher the number of pellet meals, the less equids had their ears forward (rho = −0.74; *p* = 0.037) and the more they had their ears backward (rho = 0.67, *p* < 0.001) during foraging. However, the number of meal pellets was negatively correlated with the proportion of overweight equids (rho = −0.71 *p* = 0.048).
- Housing conditions: The number of hours outdoors was negatively correlated with the proportion of equids with mostly their ears backward during foraging (rho = −0.70; *p* = 0.051). Moreover, the higher the proportion of equids spending more than 5.5 h/week outdoors, the higher the proportion of non-interactive equids in the MP test (rho = 0.82, *p* = 0.011).
- Working conditions: The higher the proportion of equids doing groundwork, the lower the proportion of equids showing no reaction in the motionless person test (rho = −0.81; *p* = 0.028); the higher the proportion of equids ridden, the lower the proportion of equids having mostly their ears forward during foraging (rho = −0.83; *p* = 0.011), presenting positive reactions in the approach–contact test (rho = −0.78; *p* = 0.023) and no reaction in the saddle test (rho = −0.74; *p* = 0.035) and in the motionless person test (rho = −0.81; *p* = 0.028); the higher the proportion of equids ridden with a bit, the higher the proportion of equids exhibiting negative behaviors during the saddle test (rho = 0.73; *p* = 0.038). However, the higher the proportion of equids doing ridden work, the lower the proportion of equids presenting at least one equipment-related injury (rho = −0.73; *p* = 0.038).

#### 6.1.6. Animal Choice and Welfare

The higher the proportion of older equids (over 16 years old), the higher the proportion of equids reacting positively in the saddle test (rho = 0.74; *p* = 0.046) and the higher the proportion of overweight equids (rho = 0.89; *p* = 0.003) were. The higher the proportion of ponies in the facilities, the lower the proportion of equids that did not show any reaction to the motionless person test was (rho = −0.77; *p* = 0.041).

The morphological type appeared also to correlate with welfare parameters, although most equids (64.0%) were dolichomorphic in all facilities. Thus, a higher proportion of brachymorphic equids was correlated with a lower proportion of unresponsive equids in the approach–contact test (rho = −0.72; *p* = 0.043). Interestingly, the higher the proportion of mesomorphic equids, the lower the proportion of equids reacting negatively to the saddle test (rho = −0.73; *p* = 0.040). On the contrary, the proportion of dolichomorphic equids tended to be positively correlated with the proportion of negative reactions at the saddle test (rho = 0.67, *p* < 0.001).

## 7. Discussion

Differences in management profiles were clear between the facilities. The results reveal three main types of management, each corresponding to two to three facilities.

Interestingly, facilities with equines exclusively engaged in EAI activities tended to house equids more in groups outdoors, whereas facilities with more equids involved in mixed activities and more conventional riding school activities housed them more in individual stalls. Logically, equid welfare profiles reflected these differences. Thus, this study showed a marked difference in the management of equids by managers of facilities primarily dedicated to RS activities compared to managers of facilities more dedicated to EAI. The last ones seemed to have more positive practices for the animals’ welfare.

Overall, in terms of management profiles, the more facilities kept their equids outdoors, with high roughage/low pellet regimen, and used groundwork or unbitted riding, the more they enhanced the chances of horses exhibiting good welfare (e.g., ears forward during foraging) and the less they expressed indicators of compromised welfare (ears backward, while foraging, unresponsiveness in human–horse tests) including responses to riding equipment. Although some of the facilities were visited at colder/wetter times (e.g., February), the temperatures remained in the thermoneutral zone for horses, and Nordic studies have shown that horses can prefer staying outdoors under cold weather unless extreme climatic constraints are encountered [65]. Therefore, even in the facilities visited at the end of winter, one turnout per week may have been insufficient, especially as this practice persisted in spring. Mixed outdoor/indoor housing may be beneficial when there are surface or weather constraints, but different studies indicate that the lack of daily turnout, allowing free movement, and ideally for several hours, is problematic for horses’ welfare, e.g., [2,37,66].

One major point is the very important impact of activity modalities on the reactions of equids towards an unfamiliar human: the more the facility uses groundwork, the more interactive are the equids, whereas the more it uses ridden work, the less equids have positive reactions towards an unfamiliar human and the more they react negatively to an approach with a saddle. These findings call for more attention to working modalities, especially in EAI where clients may not be able to control their gestures or their balance, e.g., [8]. Inappropriate hand/bit actions may generate oral lesions but also hollow necks (avoidance of bit) and, thus, back problems, e.g., [22,34,67,68,69]. Moreover, a lateral unbalance may induce unilateral pressures on the horses’ backs [70,71]. One may therefore wonder about the effect of the “double riding” (a client and professional on a horse’s back) regularly practiced in EAI. One intriguing finding is the negative correlation between the proportion of equids being ridden and the number of equipment-related lesions. On the one hand, this suggests that possibly more attention is given by the riding teacher to the appropriate fitting of the equipment by riders and/or that less-ridden equids were overweight, which may be a cause of injury, in particular at the girth level (even if just a surcingle is used).

Finally, the selection of equids appeared to be more an individual decision than a decision related to activity, but it is noteworthy that non-dolichomorphic equids, despite the latter being predominant, responded less negatively to human–horse relationship tests. The proportion of brachymorphic equids was too low for testing, but it appeared that the more mesomorphic equids there were in the facility, the less negative reactions were observed in the saddle test. Negative reactions in the saddle test may reflect negative memories of work as horses can associate objects with memories of human interactions [60,72] and/or back problems, and these reactions would be attempts to avoid being equipped and then ridden, e.g., [73]. It has been proposed that horses’ morphological type plays a role in the prevalence of back problems, with shorter backs being less at risk of being affected by repeated pressures on the back than longer backs [35]. This certainly deserves further consideration in future studies with larger samples of non-dolichomorphic equids.

A major limitation of this study remains the reduced sample of facilities (but not horses) included due to the long but objective work that was induced by such methodological choice (direct observation). Further studies should also find more facilities totally dedicated to equine-assisted interventions. This remains difficult in France at this stage, as they are rare or have a very small number of horses.

## 8. General Discussion

The results of both studies converge in that managers of facilities devoted to EAI seem more aware of the equids’ basic needs than managers of facilities primarily devoted to conventional riding school (RS) lessons. The observational study performed on eight facilities, not involved in the survey, also confirms that EAI equids remain above all equids whose welfare is impacted by both their daily life and working conditions like for other working equids. However, Study 2 suggested that equids working in both EAI and RS were more at risk of compromised welfare despite being overall managed the same way as the RS equids living on the same site. In particular, they appeared more affected still than the other horses by working modalities and more negative towards humans. This is difficult to explain. One hypothesis is that the emotional/physical strain of EAI added to the daily riding lessons, which themselves could be a source of discomfort, adding to more restricted living conditions than EAI-only equids. Another non-exclusive hypothesis is that the riding teachers in the RSs use the same modalities (more ridden and bitted work) in EAI and conventional riding lessons, here EAI clients may have disabilities that increase the equids’ discomfort at work. In France, conventional riding school activities are generally supervised by riding teachers trained for “classical riding”, whereas pure EAI activities are most often supervised by persons primarily trained for human well-being (e.g., nurse schools) and secondarily to use equids for their activity. This “cultural” difference also appears in Study 1. There were also some discrepancies between elements mentioned in the survey and field observations. For example, in the survey, half of the EAI respondents mentioned selecting equids on their morphology, mentioning a choice of more “solid” equids. However, the field study revealed that all facilities, whatever the main activity, had a majority of dolichomorphic equids. The facility managers also stated that they based their selection of animals on temperament, morphology, and relationship with humans, with more interest for relationship to human in EAI than in RS equids. The field data showed that there were equids with negative reactions (or no interaction) to an unfamiliar human in all facilities. The welfare assessment confirmed a general better welfare state in the EAI equids as compared to both RS equids and equids working in both activities. Nevertheless, they appear to be equally sensitive to riding and being bitted, which means that particular attention should be given to the particular public concern.

Part of the positive aspects of equids housed in facilities devoted to EAI was, however, that groundwork and unbitted activities were more frequent. These results constitute one of the rare evidences of correlates between human decisions in terms of the selection, management, and working conditions of the equids and their welfare state at the system level.

The correlates between working conditions and the reactions of equids to unfamiliar humans is especially interesting in the context of EAI and confirms the chronic effects of working sessions on how equids perceive humans and associated equipment in particular. This is where equid welfare and human satisfaction or even security do meet.

### 8.1. Animal Management

The management parameters described in the survey by the facility managers and confirmed to some extent by the observational study were, in terms of prevalence, different from those found more classically in other survey studies on working equids. Indeed, in other studies, the percentage of horses living permanently outdoor was between 10% [74] and 58% [75], with intermediate situations, e.g., 19% [3], 25% [76], 32% [15,77], 48% [78], whereas 54% of RS and 69% of EAI managers mentioned this practice in our studies. In other studies, owners reported that between 44% [15] and 47% [79] of the horses lived permanently in groups, whereas our study reported 54% and 59%, respectively. In terms of feeding, on the contrary, the proportions found here (permanent access to hay for EAI equids, 59.4%; more than 9 kg of hay per day for RS equids, 54.5%) were quite similar to those found by Hockenhull and Creighton [15] and by Mellor and collaborators [75] with, respectively, 50% and 87% of horses having free access to hay and 60% of horses having at least one meal of pellets per day. Larsson and Müller [76] had reported only 25% of horses with ad libitum access to hay and 74% with pellets. Leme and collaborators [74] found that over 90% of horses were fed pellets in riding schools. It would, therefore, appear that most of the facilities managers that participated in our survey reported with a more positive management in terms of welfare than was more commonly seen. The observational results differed also from those observed in populations of more conventional riding school or sport facilities [2,37] where the equids were almost all in stalls with little daily exercise and less opportunity for social contact, as well as generally fed less hay and more pellet meals. These differences could arise from sampling bias. One could argue that our study participants (both Studies 1 and 2) were interested in scientific studies on horses and, therefore, more aware of recommended management practices for animal welfare. It is also possible that better animal management was performed in EAI than in equestrian facilities using a more traditional management. There is still a low number of studies addressing the management of equids in EAI programs [9], which means further studies are needed to see whether our findings are of general value.

Indeed, the results of the questionnaire and the field study showed differences according to the predominant activities of the facilities. The managers of facilities dedicated mainly to riding lessons had a training background, or a perception of animal management, that differed greatly from that of people who were more centered on EAI. It has already been shown that in horse riding facilities, some people did not change their practices because of pressure from others in the community to maintain more “traditional” management methods [18] and that even when people are informed about what is good for the animal, they do not necessarily put it into practice for their own horses, showing a difference between theoretical knowledge and its procedural application [16]. In addition, it has been shown that some riding magazines aimed at the general public may disseminate erroneous information, particularly on “good” management practices for horses [17,80]. Finally, it has also been shown that false beliefs can exist among professionals. In particular, it has been shown that professionals attribute behavioral differences and needs to different equid sexes, which could lead to differences in management [24,30]. Overall, the representation of good management practices seems to be particularly influenced by the “equestrian culture”, which differs according to the managers’ equestrian background.

### 8.2. Criteria for the Selection of Animals

The questionnaires showed that, among the criteria for choosing an animal, personality was the most cited, followed by the physical characteristics of the animal (morphology) and then the relationship with humans by RS managers, whereas EAI managers put much more emphasis on the relationship of equids with humans. The level of education and the health and background were next at roughly equal levels. These results were similar to those found by Heydemann and Grosbois [81] for horses and Rankins and collaborators [13] for EAI equids, whereas sex, namely, geldings, were preferred in Watson et al.’s study [24] on US EAI equids. Sex was not mentioned as important in our survey, and there seemed to be some individual preferences of managers for one sex or one type of equid, but these could be the opposite between managers. There was also no consensual choice for one sex or another in Study 2: Some managers preferred to use mares (e.g., facility 1), others geldings (e.g., facility 3), whereas others had no preference. When questioned, the manager who preferred mares said it was because they were “gentler” than geldings, while the manager who preferred geldings said that because they had no sexual cycles, their behavior was more stable over time (pers. obs.). According to the managers, the choice of having more ponies or horses in facilities was mostly related to the most represented age category amongst the clients: the more there were children and young teenagers, the more they used ponies. The fact that a greater number of EAI facilities managers stated that they had some selection criteria seems consistent with the fact that it is particularly recommended to select horses for EAI [7,33]. However, a recent study found that there was no difference in temperament or relationship to humans between animals that had and had not been selected by professionals for EAI [82]. These results were consistent with those of Anderson and collaborators [33] and Minero and collaborators [83] who found no difference between EAI and RS equines in temperament tests. EAI facilities managers reported more interest in the relationship with humans, but in all the facilities studied here, between 10 and 50% of the animals showed negative reactions to humans during the tests. Furthermore, it has recently been shown that EAI horses are less responsive to humans than RS or sport equines [10,11,82], which may be a neutral response or reflect a rather negative perception of humans, e.g., [36].

Finally, with regard to the selection on morphological criteria, most managers mentioned a preference for “solid” equids, i.e., of the brachymorphic type. However, the field observations showed, like in conventional riding schools, a high proportion of dolichomorphic equids, see also [2]. This may be of high importance, especially for EAI where a large diversity of clients may ride, as brachymorphic equids could be less susceptible to having back problems [35]. Back problems are one of the most prevalent problems in ridden horses, see review [34], and was mentioned as one of the most prevalent problems in EAI equids in Watson et al.’s study [24].

### 8.3. Correlates between Human Decisions and Equids’ Welfare

Overall, our findings converged with most earlier studies showing that permanent group outdoor or partial housing, as well as continuous access to roughage, correlated with better welfare states, e.g., [2,3,37]. Ad libitum access to roughage is associated with less stereotypic behaviors and colic and more positive behaviors towards conspecifics and humans [2,54,66,84,85,86]. Respondents mentioned a lower prevalence of colic and ulcers in their EAI equids in Watson et al.’s study [24], maybe because they also had this feeding strategy and stereotypies. On the other hand, our study converges also with earlier findings in showing that horses fed pellet meals had more negative welfare indicators, while other studies have shown positive correlations with the rate of stereotypies [2,87,88], and significant digestive problems [89]. According to Ermers and collaborators [90], horses should be able to forage for at least 8 h a day, and horses on forage-only diets have a greater microbial diversity.

The more striking and original findings of our observational study are probably the clear correlates between working modalities and equids’ welfare. Several earlier studies had already shown that if the number of hours does not really impact the welfare states of the animals, the modalities of work do [2,34,67,91]. Thus, the rider’s position, horse posture, or the equipment [71,92,93] have chronic effects (i.e., outside working sessions) on the ridden equids. In our study, facilities where EAI equids performed mostly groundwork had a higher prevalence of interactive equids during the human–horse relationship tests, whereas a higher prevalence of ridden equids was correlated with less positive welfare indicators and unresponsiveness in the saddle test. There was a fair proportion of equids working bitless (almost 30% as compared to 5% in Luke et al.’s study [22], especially in the facilities devoted to EAI. Remarkably, the equids doing bitted work showed more negative reactions in the saddle test. The saddle test is likely to evoke horse’s associative memories of work [60], in which case, negative reactions may reflect the aversion of these equids for working activities. There is growing evidence that the use of the bit, especially by inexperienced riders, is associated with a higher prevalence of lesions in the mouth, on the commissure, and may induce breathing difficulties and back problems through head raising (i.e., hollowing the neck and beck) to avoid the pain [2,22,34,44,67,68,69,93]. In a survey study based on horse owners’ responses on 66 ridden horses, Cook and Kibler [94] found that all but one exhibited bit aversion and that the first examples of evidence of pain were “hating the bit”, fright and anxiety, a stiff neck, and loss of control. In the same line, comparisons of the same horses, including EAI horses ridden either with a bitted bridle or a bitless bridle, have shown that there were more head raising and other undesirable behaviors when they were ridden with a bit [95,96].

Precisely, in our observational study, the more the facility was involved in EAI, the less equids had a hollow or flat neck, reflective of tensions in the spine [44]. On the other hand, the more the facilities used equids for riding lessons, the less interactive their equids were in the saddle test. Moreover, the more equids were involved in mixed activities, the more stereotypic behaviors they performed outside work and the more they showed negative reactions in the saddle test. Different studies have reported important welfare problems in conventional riding schools but also differences between facilities [2,5,39,46,49]. This may be due in particular to working modalities such as riding techniques, which vary according to the riding teacher’s perception of teaching priorities (riders’ positions or control of the horse) [2,34,67]. Given the large discrepancy observed in our survey in the perception of management between EAI and RS managers, it is likely that such differences extend to working modalities. Thus, Rankins and collaborators [13] mentioned that many EAI respondents indicated using positive reinforcement in training their horses, which is unusual still in the general equestrian world, e.g., [97,98]. In our observational study, none of the facilities used positive reinforcement despite it being recommended by the IAHAIO [7]. There may be both equestrian and national cultures influencing the choice of training techniques.

Interestingly, in our study, facilities with higher proportions of horses working both in EAI and RS appeared to have the most compromised equid welfare. However, in both the survey and the observational study, managers of facilities with such mixed activities seemed to be more convergent with managers of RS facilities. It may be then that there is more ridden and bitted work in these facilities even for EAI horses, which, given the unbalance and possible inappropriate hand and leg actions of persons with disabilities, may induce still more pain during the sessions. Both chronic consequences (see higher prevalence of stereotypic horses) and aversion for work (see higher proportion of equids with negative reactions to the saddle test) may then be expected. Interestingly, inappropriate grooming actions of persons with mental disabilities might explain the tactile hypersensitivity of EAI equids as compared to RS equids [99].

Finally, one common feature to all facilities, in contradiction with the survey, is the high prevalence of dolichomorphic equids, which are supposed to be more at risk for back problems [35]. In our study, the more the facility had brachymorphic equids, the more the equids were interactive in the approach–contact test and the more mesomorphic horses showed fewer negative reactions in the saddle test.

Managing EAI facilities involves, therefore, an array of decisions that each may have important consequences, and the additions of all these decisions may have additive effects. It is clear that the managers’ decisions are influenced by his/her training and social background. Obviously more mixing between equestrian cultures would be a good way of enlarging an individual’s view of good management practices.

## 9. Conclusions

This study, based on two different but complementary approaches, is one of the first to consider both the human and equine points of view on the importance of living and working conditions for equine (chronic) welfare, especially where equine-assisted interventions are involved. The results showed that EAI equids are the first of all equids with the same adaptations for continuous feeding, free movement, and social life and that, therefore, these aspects have to be considered beyond the activity itself. While further studies with larger samples are still needed, the results also emphasized the great importance of working modalities for horses’ welfare and perception of humans, which is of the highest importance for EAI. One major finding was also the importance of managers’ culture, showing how important it is that training includes appropriate information on all these aspects.

## Figures and Tables

**Table 1:** Extraction from the survey as an example.

In most cases, where do the horses/ponies (excluding Shetlands) that work in riding lessons live? □ In stall (<3 h/day of free exercise in the pasture or paddock) □ Mixed, i.e., stall and pasture or paddock (≥3 h/day of free exercise in the pasture or paddock) □ In pasture or paddock all the time If stall or mixed When horses/ponies (excluding Shetlands) working in riding lessons are indoors, what type of bedding do they have? □ Straw □ Shavings □ Other, please specify ………………………………… In most cases, when horses/ponies (excluding Shetlands) working in riding lessons are stalled, what is their situation? □ Single (individual stall) □ In group (collective housing) If mixed or pasture/paddock In most cases, when horses/ponies (excluding Shetlands) working in riding lessons are in the pasture or paddock, what is their situation? □ Single □ In group In most cases, how many meals of pellets or grain do the horses/ponies (excluding Shetlands) working in riding lessons have every day? □ 0 meal per day □ 1 meal per day □ 2 meals per day □ 3 or more meals per day In most cases, how many meals of hay per day do the horses/ponies (excluding Shetlands) working in riding lessons have? □ None □ Once a day □ Twice a day □ Permanent access In most cases, what amount of hay (in kilograms per horse) do the horses/ponies (excluding Shetlands) working in riding lessons have per day? □ 0 kg to 3 kg □ 3 kg to 9 kg □ more than 9 kg

**Table 2:** Number of facilities according to the activities proposed.

Activities	Number of Facilities
Riding school lessons	5
Riding school lessons and EAI	4
Riding school lessons, EAI, and Boarding	14
Riding school lessons and Boarding	11
Riding school lessons, EAI, and Tourism	3
Riding school lessons, EAI, Tourism, and Boarding	4
Riding school lessons, Tourism, and Boarding	3
EAI	4
EAI and Boarding	2
EAI, Tourism, and Boarding	1

**Table 3:** Information about management of equids according to their activities. In italic, the detailed quantity of meals of pellets or cereals received per day.

	Information about Equids’ Management			
	Riding School Lessons		Equine-Assisted Interventions	
	%	N	%	N
Permanent pasture or paddock	54.5%	24	68.8%	22
Paddock and stall	27.3%	12	12.5%	4
Permanent stall housing	18.2%	8	18.8%	6
Always in group	54.5%	24	59.4%	19
>3 h/day in group	25.0%	11	12.5%	4
Permanent single housing	20.5%	9	28.1%	9
>9 kg of hay/day	54.5%	24	40.6%	13
3 kg to 9 kg of hay/day	36.4%	16	46.9%	15
<3 kg of hay/day	9.1%	4	12.5%	4
No pellets/cereals	20.5%	9	40.6%	13
Pellets or cereals	79.5%	35	59.4%	19
*1 meal per day*	*18.2%*	*8*	*21.9%*	*7*
*2 meals per day*	*36.4%*	*16*	*12.5%*	*4*
*3 or more meals per day*	*25.0%*	*11*	*25.0%*	*8*

**Table 4:** Characteristics of the 8 facilities included. Pasture is an area of ground covered with grass to feed on; paddock is a small field, with little or no grass in which horses are temporarily kept. NA corresponds to “not applicable”.

Facility Number	Equid Number	Mean Age (yo ± SE)	Outdoor Housing (Free Turnout)			Indoor Housing				Roughage (Hay/Grass)		Pellets		Working per Week (Hours ± SE)
			Outside Housing per Day (Hours)	Alone or Group	Type	Hours per Day	Single or Group	Type	Bedding	Total Amount per Day	Number of Meals per Day	Total Amount per Day (Liters)	Number of Meals per Day	
1	5	10 ± 6	24	Group	Pasture	0	N/A	N/A	N/A	N/A	Ad libitum	N/A	N/A	3 ± 1.9
2	11	16 ± 6	12	Group	Pasture	12	Group	Communal stall	N/A	Ad libitum		N/A	N/A	1.25 ± 0.5
3	20	14 ± 6	24	Group	Pasture	0	N/A	N/A	N/A	Ad libitum		0.25	1	4.4 ± 1.6
4	34	15 ± 5	22 ± 2	Group	Pasture	1 ± 2	No information	Single or communal stall	Straw	Ad libitum		N/A	N/A	11 ± 3
5	25	11 ± 3	17 ± 9	Group	Paddock	7 ± 10	Single	Single stall	Straw	Ad libitum		4	2 or 3	10 ± 2.8
6	19	14 ± 6	21 ± 5	Group	Pasture	3 ± 5	Single	Single stall	Sawdust	Ad libitum		0.5	2	8.6 ± 2.3
7	27	13 ± 8	1 ± 0.3	Group	Paddock	23 ± 0.7	No information	Single or communal stall	Straw	Ad libitum		3.7	1 to 3	3.2 ± 2.3
8	33	15 ± 5	0.8 (1 day of 6 h once a week)	NA	NA	24 (6 days a week) + 18 once a week	Single	Single or communalstall	Wood shavings	2 to 6 kgs	3	0.5	2	No quantified information

## Data Availability

The raw data supporting the conclusions of this article will be made available by the authors on request.

## Supplementary Materials

The following supporting information can be downloaded at https://www.mdpi.com/article/10.3390/ani14172548/s1. Table S1: Survey; Table S2: Spearman correlation coefficients between welfare indicators and activity, management, and animal choice parameters. Spearman correlation test (rho). N = 8 facilities. For significance: rho *p* < 0.07: rho, *p* < 0.05.
