# Supplementary material for: From Human Perception of Good Practices to Horse (Equus Caballus) Welfare: Example of Equine-Assisted Activities
Source: Animals (Basel). 2024 Sep 2;14(17):2548. doi: 10.3390/ani14172548 (PMC11393981; doi:10.3390/ani14172548)
Supplement: Supplementary file 1 [file animals-14-02548-s001.zip › animals-3093477-supplementary.pdf]

Suppl. Table S1. Survey

This questionnaire is part of a study conducted by the EthoS Laboratory at the University of Rennes 1 on horses present in equine facilities. Its purpose is to gather information on the management practices of horses in various facilities across metropolitan France. This questionnaire is intended for anyone currently holding responsibilities in an equine facility in France. The estimated time for completing this questionnaire is between 10 and 20 minutes. There are no right or wrong answers, and all your responses will be anonymized. All questions in the questionnaire are mandatory.

We appreciate your willingness to participate in our study and thank you in advance for the time you will dedicate to this questionnaire. For any questions related to this survey, please contact Noémie Lerch, EthoS PhD student (*email link*).

PART 1

Be careful: For all questions, we distinguish Shetlands from other ponies

Part A : introduction

A1- Currently, how many horses and ponies (excluding Shetlands) are present in your facility?

Give number

A2- Currently, do you have Shetlands in your facility?

O Yes O No

A2.1- If yes, how many Shetlands are present in your facility?

Give number

A3- What activities from this list do you develop in your equine facility?

O Riding lessons

O Equestrian tourism (outdoors leisure horse riding, for at least 50 days of riding/year)

O Equine-assisted interventions (i.e. activities with people with disabilities such as equine-assisted therapy, riding for the disabled)

O Horse/Pony boarding (i.e. private horses/ponies kept on the facility)

PART B : If Riding Lesson (excluding Shetlands)

B01- What are your criteria for identifying a horse, a pony, or a Shetland suitable for this activity (riding lessons)?

Give free response

B02- How many horses and ponies (excluding Shetlands) are involved in riding lessons?

Give number

B03- Generally, how many riding lesson sessions do horses and ponies (excluding Shetlands) have per week?

Give number

B04- Generally, how long do the riding lesson sessions involving horses and ponies (excluding Shetlands) last?

Give time in hours or minutes

B05- Give comments if necessary

Give free response

B06- Where do the horses/ponies (excluding Shetlands) working in the context of riding lessons mostly live?

O In a box (<3h/day of free exercise in the pasture or paddock)

O Mixed, i.e. in box and pasture or paddock (≥ 3h/day of free exercise in the pasture or paddock)

O In the pasture or paddock all the time

B07- Give comments if necessary

Give free response

If B06 answer is « Box or Mixed »

B061- When horses/ponies (excluding Shetlands) working in the context of riding lessons are inside, what is the composition of their bedding?

O Straw

O Wood shavings

O Other, please specify

B062- In the majority, when horses/ponies (excluding Shetlands) working in the context of riding lessons are in a box, what is their situation?

O Alone (individual box)

O In a group (collective stable)

B063- Give comments if necessary

Give free response

If B06 answer is « Mixed or Pasture/Paddock »

B064- In the majority, when horses/ponies (excluding Shetlands) working in the context of riding lessons are in the pasture or paddock, what is their situation?

O Alone

O In a group

B065- Give comments if necessary

Give free response

B08- In the majority, how many meals of pellets do horses/ponies (excluding Shetlands) working in the context of riding lessons have each day?

O 0 meals per day

O 1 meal per day

O 2 meals per day

O 3 meals or more per day

B09- Give comments if necessary

Give free response

B10- In the majority, how many meals of hay do horses/ponies (excluding Shetlands) working in the context of riding lessons have each day?

O 0 times per day

O 1 time per day

O 2 times per day

O Permanent access

B11- Give comments if necessary

Give free response

B12- In the majority, how many kilograms of hay do horses/ponies (excluding Shetlands) working in the context of riding lessons have each day (per horse)?

O 0kg to 3kg

O 3kg to 9kg

O more than 9kg

B13- Give comments if necessary

Give free response

<u>PART C : If Riding Lesson (for Shetlands only)</u>

C01- Currently, are Shetlands involved in riding lessons?

O Yes O No

If yes

C02- How many Shetlands are involved in riding lessons?

Give number

C03- Generally, how many riding lesson sessions do Shetlands have per week?

Give number

C04- Generally, how long do riding lesson sessions involving Shetlands last?

Give time in hours or minutes

C05- Give comments if necessary

Give free response

C06- In the majority, where do Shetlands working in the context of riding lessons live?

O In a box (<3h/day of free exercise in the pasture or paddock)

O Mixed, meaning box and pasture or paddock (≥ 3h/day of free exercise in the pasture or paddock)

O In the pasture or paddock all the time

C07- Give comments if necessary

Give free response

If answer of C06 is « Box or Mixed »

C061- When Shetlands working in the context of riding lessons are indoors, what is the composition of their bedding?

O Straw

O Wood shavings

O Other, *please specify*

C062- In the majority, for Shetlands working in the context of riding lessons that are in a box, what is their situation?

O Alone (individual box)

O In a group (stable)

C063- Give comments if necessary

Give free response

If answer of C06 is « Mixed or Pasture/Paddock »

C064- In the majority, when Shetlands working in the context of riding lessons live in the pasture or paddock, what is their situation?

O Alone

O In a group

C065- Give comments if necessary

Give free response

C08- In the majority, how many meals of pellets do Shetlands working in the context of riding lessons have each day?

O 0 meals per day

O 1 meal per day

O 2 meals per day

O 3 meals or more per day

C09- Give comments if necessary

Give free response

C10- In the majority, how many meals of hay do Shetlands working in the context of riding lessons have each day?

O 0 times per day

O 1 time per day

O 2 times per day

O Permanent access

C11- Give comments if necessary

Give free response

C12- In the majority, how many kilograms of hay do Shetlands working in the context of riding lessons have each day (per horse)?

O 0kg to 3kg

O 3kg to 9kg

O more than 9kg

C13- Give comments if necessary

Give free response

PART D : If Equestrian Tourism (excluding Shetlands)

D01- What are your criteria for identifying a horse, pony, or Shetland suitable for equestrian tourism?

Give free response

D02- How many horses and ponies (excluding Shetlands) are involved in tourism activities?

Give number

D03- Generally, how many hours of outdoor riding do horses and ponies (excluding Shetlands) working in tourism activities do per week?

Give time in hours

D04- Give comments if necessary

Give free response

D05- In the majority, where do horses/ponies (excluding Shetlands) working in tourism activities live?

O In a box (<3h/day of free exercise in the pasture or paddock)

O Mixed, i.e. box and pasture or paddock (≥ 3h/day of free exercise in the pasture or paddock)

O In the pasture or paddock all the time

D06- Give comments if necessary

Give free response

If answer to DO5 is « Box or Mixed »

D051- When horses/ponies (excluding Shetlands) working in tourism activities are indoors, what is the composition of their bedding?

O Straw

O Wood shavings

O Other, *please specify*

D052- In the majority, when horses/ponies (excluding Shetlands) working in tourism activities are in a box, what is their situation?

O Alone (individual box)

O In a group (stable)

D053- Give comments if necessary

Give free response

If answer to DO5 is « Mixed or Pasture/Paddock »

D054- In the majority, when horses/ponies (excluding Shetlands) working in tourism activities are in the pasture or paddock, what is their situation?

O Alone

O In a group

D055- Give comments if necessary

Give free response

D07- In the majority, how many meals of pellets do horses/ponies (excluding Shetlands) working in tourism have each day?

O 0 meals per day

O 1 meal per day

O 2 meals per day

O 3 meals or more per day

D08- Give comments if necessary

Give free response

D09- In the majority, how many meals of hay do horses/ponies (excluding Shetlands) working in tourism have each day?

O 0 times per day

O 1 time per day

O 2 times per day O Permanent access

D10- Give comments if necessary

Give free response

D11- In the majority, how many kilograms of hay do horses/ponies (excluding Shetlands) working in tourism have each day (per horse)?

O 0kg to 3kg

O 3kg to 9kg

O more than 9kg

D12- Give comments if necessary

Give free response

<u>PART E: If Equestrian Tourism (only for Shetlands)</u>

E01- Currently, are Shetlands involved in tourism activities?

O Yes

O No

If yes

E02- How many Shetlands are involved in tourism activities?

Give number

E03- Generally, how many hours of outdoor riding do Shetlands working in tourism activities do per week?

Give time in hours

E04- Give comments if necessary

Give free response

E05- In the majority, where do Shetlands working in tourism activities live?

O In a box (<3h/day of free exercise in the pasture or paddock)

O Mixed, i.e. box and pasture or paddock (≥ 3h/day of free exercise in the pasture or paddock)

O In the pasture or paddock all the time **E06-** Comments: [Space for comments]

If answer to E05 is « Box or Mixed »

E051- When Shetlands working in tourism activities are indoors, what is the composition of their bedding?

O Straw

O Wood shavings

O Other*, please specify*

E052- In the majority, when Shetlands working in tourism activities are in box, what is their situation?

OAlone (individual box)

O In a group (stable)

E053- Give comments if necessary

Give free response

If answer to E05 is « Mixed or Pasture/Paddock »

E054- In the majority, when Shetlands working in tourism activities are in the pasture or paddock, what is their situation?

O Alone

O In a group

E055- Give comments if necessary

Give free response

E07- In the majority, how many meals of pellets do Shetlands working in tourism have each day?

O 0 meals per day

O 1 meal per day

O 2 meals per day

O 3 meals or more per day

E08- Give comments if necessary

Give free response

E09- In the majority, how many meals of hay do Shetlands working in tourism have each day?

O 0 times per day

O 1 time per day

O 2 times per day

O Permanent access

E10- Give comments if necessary

Give free response

E11- In the majority, how many kilograms of hay do Shetlands working in tourism have each day (per horse)?

O 0kg to 3kg

O 3kg to 9kg

O more than 9kg

E12- Give comments if necessary

Give free response

Part F : if Equine Assisted Interventions or EAI (excepted for Shetlands)

F01- What are your criteria for identifying a horse, pony, or Shetland suitable for EAI?

Give free response

F02- How many horses and ponies (excluding Shetlands) are involved in EAI?

Give number

F03- Generally, how many EAI sessions do horses and ponies (excluding Shetlands) participate in per week?

Give number

F04- Generally, how long do EAI sessions involving horses and ponies (excluding Shetlands) last?

Give time in hours or minutes

F05- Give comments if necessary

Give free response

F06- In the majority, where do horses/ponies (excluding Shetlands) working in EAI activities live?

O In a box (<3h/day of free exercise in the pasture or paddock)

O Mixed, i.e. box and pasture or paddock (≥ 3h/day of free exercise in the pasture or paddock)

O In the pasture or paddock all the time

F07- Give comments if necessary

Give free response

If answer to F06 is « Box or Mixed »

F061- When horses/ponies (excluding Shetlands) in EAI activities are indoors, what is the composition of their bedding?

O Straw

O Wood shavings

O Other, *please specify*

F062- In the majority, when horses/ponies (excluding Shetlands) working in EAI activities are in a box, what is their situation?

O Alone (individual box)

O In a group (stable)

F063- Give comments if necessary

Give free response

If answer to F06 is « Mixed or Pasture/Paddock »

F064- In the majority, when horses/ponies (excluding Shetlands) working in EAI activities are in the pasture or paddock, what is their situation?

O Alone

O In a group

F065- Give comments if necessary

Give free response

F08- In the majority, how many meals of pellets do horses/ponies (excluding Shetlands) working in EAI have each day?

O 0 meals per day

O 1 meal per day

O 2 meals per day

O 3 meals or more per day

F09- Give comments if necessary

Give free response

F10- In the majority, how many meals of hay do horses/ponies (excluding Shetlands) working in EAI have each day?

O 0 times per day

O 1 time per day

O 2 times per day

O Permanent access

F11- Give comments if necessary

Give free response

F12- In the majority, how many kilograms of hay do horses/ponies (excluding Shetlands) working in EAI have each day (per horse)?

O 0kg to 3kg

O 3kg to 9kg

O more than 9kg

F13- Give comments if necessary

Give free response

<u>Part G : if Equine Assisted Interventions or EAI (only for Shetlands)</u>

G01- Currently, are Shetlands involved in EAI activities?

O Yes

O No

If yes

G02- How many Shetlands are involved in EAI activities?

Give number

G03- Generally, how many EAI sessions do Shetlands participate in per week?

Give number

G04- Generally, how long do EAI sessions involving Shetlands last?

Give time in hours or minutes

G05- Give comments if necessary

Give free response

G06- In the majority, where do Shetlands working in EAI activities live?

O In a box (<3h/day of free exercise in the pasture or paddock)

O Mixed, i.e. box and pasture or paddock (≥ 3h/day of free exercise in the pasture or paddock)

O In the pasture or paddock all the time

G07- Give comments if necessary

Give free response

If answer to G06 is « Box or Mixed »

G061- When Shetlands in EAI activities are indoors, what is the composition of their bedding?

O Straw

O Wood shavings

O Other, *please specify*

G062- In the majority, when Shetlands working in EAI activities are in box, what is their situation?

O Alone (individual box)

O In a group (stable)

G063- Give comments if necessary

Give free response

If answer to G06 is « Mixed or Pasture/Paddock »

G064- In the majority, when Shetlands working in EAI activities are in the pasture or paddock, what is their situation?

O Alone

O In a group

G065- Give comments if necessary

Give free response

G08- In the majority, how many meals of pellets do Shetlands working in EAI have each day?

O 0 meals per day

O 1 meal per day

O 2 meals per day

O 3 meals or more per day

G09- Give comments if necessary

Give free response

G10- In the majority, how many meals of hay do Shetlands working in EAI have each day?

O 0 times per day

O 1 time per day

O 2 times per day

O Permanent access

G11- Give comments if necessary

Give free response

G12- In the majority, how many kilograms of hay do Shetlands working in EAI have each day (per horse)?

O 0kg to 3kg

O 3kg to 9kg

O more than 9kg

G13- Give comments if necessary

Give free response

Part H : If Boarding (excepted Shetlands)

H01- Do you select horses, ponies or Shetlands owned by individuals for boarding in your facility?

O Yes O No

If yes

H02- What are your selection criteria?

Give free response

H03- How many horses and ponies (excluding Shetlands) owned by individuals are boarded in your facility?

Give number

H04- To your knowledge, how many work sessions do horses and ponies (excluding Shetlands) in boarding have per week on average?

Give number or

O Don't know

H05- To your knowledge, how long do work sessions for horses and ponies (excluding Shetlands) in boarding last on average?

Give time in hours or minutes or

O Don't know

H06- Give comments if necessary

Give free response

H07- In the majority, where do horses/ponies (excluding Shetlands) in boarding live in your facility?

O In a box (<3h/day of free exercise in the pasture or paddock)

O Mixed, i.e. box and pasture or paddock (≥ 3h/day of free exercise in the pasture or paddock)

O In the pasture or paddock all the time

H08- Give comments if necessary

Give free response

If answer H07 is « Box or Mixed »

H071- When horses/ponies (excluding Shetlands) in boarding are indoors, what is the composition of their bedding?

O Straw

O Wood shavings

O Other, *please specify*

H072- In the majority, when horses/ponies (excluding Shetlands) in boarding are in box, what is their situation?

O Alone (individual box)

O In a group (stable)

H073- Give comments if necessary

Give free response

If answer H07 is « Mixed or Pasture/Paddock »

H074- In the majority, when horses/ponies (excluding Shetlands) in boarding are in the pasture or paddock, what is their situation?

O Alone

O In a group

H075- ***Give comments if necessary***

Give free response

H09- In the majority, how many meals of pellets do horses/ponies (excluding Shetlands) in boarding have each day?

O 0 meals per day

O 1 meal per day

O 2 meals per day

O 3 meals or more per day

H010- Give comments if necessary

Give free response

H11- In the majority, how many meals of hay do horses/ponies (excluding Shetlands) in boarding have each day?

O 0 times per day

O 1 time per day

O 2 times per day

O Permanent access

H12- Give comments if necessary

Give free response

H13- In the majority, how many kilograms of hay do horses/ponies (excluding Shetlands) in boarding have each day (per horse)?

O 0kg to 3kg

O 3kg to 9kg

O more than 9kg

H14- Give comments if necessary

Give free response

<u>Part I : If Boarding (only for Shetlands)</u>

I01- Currently, are Shetlands in boarding in your facility?

O Yes

O No

If yes

I02- How many Shetlands are in boarding in your facility?

Give number

I03- To your knowledge, how many work sessions do Shetlands in boarding have per week on average?

Give number, or

O Don't know

I04- To your knowledge, how long do work sessions involving Shetlands in boarding last on average?

Give time in hours or minutes, or

O Don't know

I05- Give comments if necessary

Give free response

I06- In the majority, where do Shetlands in boarding live in your facility?

O In a box (<3h/day of free exercise in the pasture or paddock)

O Mixed, meaning box and pasture or paddock (≥ 3h/day of free exercise in the pasture or paddock)

O In the pasture or paddock all the time

I07- Give comments if necessary

Give free response

If answer of I06 is « Box or Mixed »

I061- When Shetlands in boarding are indoors, what is the composition of their bedding?

O Straw

O Wood shavings

O Other, *please specify*

I062- In the majority, when Shetlands in boarding are in a box, what is their situation?

O Alone (individual box)

O In a group (stable)

I063- Give comments if necessary

Give free response

If answer of I06 is « Mixed or Pasture/Paddock »

I064- In the majority, when Shetlands in boarding are in the pasture or paddock, what is their situation?

O Alone

O In a group

I065- Give comments if necessary

Give free response

I08- In the majority, how many meals of pellets do Shetlands in boarding have each day?

O 0 meals per day

O 1 meal per day

O 2 meals per day

O 3 meals or more per day

I09- Give comments if necessary

Give free response

I10- In the majority, how many meals of hay do Shetlands in boarding have each day?

O 0 times per day

O 1 time per day

O 2 times per day

O Permanent access

I11- Give comments if necessary

Give free response

I12- In the majority, how many kilograms of hay do Shetlands in boarding have each day (per horse)?

O 0kg to 3kg

O 3kg to 9kg

O more than 9kg

I13- Give comments if necessary

Give free response

PART 2

All the information below will be anonymized upon receipt of the questionnaire.

Equestrian Center Name:

Give free response

O Prefer not to answer

Postal Code of the Equestrian Center:

Give free response

O Prefer not to answer

If Prefer not to answer

Department of the Equestrian Center:

Gender:

O Male

O Female

O Other

O Prefer not to answer

Year of Birth:

Give free response

O Prefer not to answer

Role/Position in the Equestrian Center:

Give free response

O Prefer not to answer

Do you wish to be informed of the study results?

O No

O Yes, please specify your email address

Thank you for the time you dedicated to answering this questionnaire. For any questions, you can write to Noémie Lerch (email link), Martine Hausberger and Marine Grandgeorge

Suppl. Table S2. Spearman correlation coefficients between welfare indicators and activity, management, and animal choice parameters. Spearman correlation test (rho).N=8 facilities. For

		Stereotypy	App-C negative	Saddle negative	Neck problem	Ears backward	Lesion	Overweight	Ears forward	Saddle positive	App-C positive	0react MP	0rect App-C	0react Saddle
**Animal selection**	**Brachymorph**	-0,37	-0,10	-0,22	-0,34	-0,01	0,01	0,04	-0,10	0,10	0,25	-0,15	**-0,72**	0,06
	**Mesomorph**	-0,32	-0,41	**-0,73**	-0,36	-0,41	-0,01	0,28	0,47	0,07	0,05	0,05	-0,14	0,31
	**Dolichomorph**	0,51	0,25	***0,67***	0,50	0,05	0,19	-0,29	-0,14	-0,05	-0,24	-0,43	0,57	-0,40
	**Mare**	-0,24	0,07	-0,18	-0,24	0,06	-0,37	0,30	-0,46	0,69	-0,45	-0,61	-0,55	-0,60
	**Pony**	0,08	0,59	0,47	-0,06	-0,23	0,05	0,16	0,02	0,24	-0,05	**-0,77**	-0,21	**-0,76**
	**>16 years old**	-0,57	0,56	-0,36	-0,29	-0,23	-0,53	**0,89**	-0,02	**0,74**	-0,33	-0,34	-0,36	-0,60
**Management**	**Roughage**	-0,20	-0,25	-0,06	0,00	-0,41	0,44	-0,19	0,06	0,13	0,13	**-0,80**	-0,38	-0,13
	**Pellet meals**	0,42	-0,21	0,39	0,34	***0,67***	0,00	**-0,71**	***-0,70***	-0,41	-0,22	0,48	0,41	0,29
	**h/day outside**	-0,63	0,30	-0,31	-0,57	**-0,74**	0,28	0,16	0,36	-0,11	0,26	-0,52	-0,56	-0,16
	**Work >5.5h/week**	-0,04	0,19	0,20	-0,18	-0,59	0,44	-0,03	0,42	-0,02	0,29	**-0,84**	-0,34	-0,38
	**Riding**	0,24	0,34	0,21	-0,22	0,59	**-0,73**	0,13	**-0,83**	0,49	**-0,78**	-0,14	-0,16	**-0,74**
	**Groundwork**	0,19	0,22	0,29	-0,10	-0,27	0,10	0,02	0,07	0,24	-0,15	**-0,81**	-0,17	-0,66
	**Bit**	0,54	-0,02	**0,73**	0,51	-0,02	0,56	-0,52	0,12	-0,30	0,30	-0,28	0,30	-0,07
**Activity**	**RS**	-0,43	-0,44	-0,35	0,43	-0,41	0,63	-0,13	0,48	-0,36	0,60	0,25	0,24	**0,88**
	**EAI-RS**	**0,92**	0,24	**0,90**	0,27	0,55	-0,10	-0,44	-0,33	-0,19	-0,23	0,07	0,43	-0,42
	**EAI**	-0,47	0,07	-0,65	**-0,73**	0,02	-0,54	0,41	-0,13	0,19	-0,24	0,16	-0,50	-0,02

significance: *rho p<0.07:* ***rho****, p<0.05*
